# A Local Search Algorithm with Vertex Weighting Strategy and Two-Level Configuration Checking for the Minimum Connected Dominating Set Problem

**DOI:** 10.3390/biomimetics9070429

**Published:** 2024-07-15

**Authors:** Ruizhi Li, Jintao He, Shangqiong Liu, Shuli Hu, Minghao Yin

**Affiliations:** 1 School of Management Science and Information Engineering, Jilin University of Finance and Economics, Changchun 130117, China; lirz111@nenu.edu.cn (R.L.); hejintao1903@163.com (J.H.); 18238950057@163.com (S.L.); 2 Business Big Data Research Center of Jilin Province, Changchun 130117, China; 3 Key Laboratory of Symbolic Computation and Knowledge Engineering of Ministry of Education, Jilin University, Changchun 130012, China; 4 School of Computer Science and Information Technology, Northeast Normal University, Changchun 130117, China

**Keywords:** perturbation strategy, simplification rule, scoring functions, minimum connected dominating set

## Abstract

The minimum connected dominating set problem is a combinatorial optimization problem with a wide range of applications in many fields. We propose an efficient local search algorithm to solve this problem. In this work, first, we adopt a new initial solution construction method based on three simplification rules. This method can reduce the size of the original graph and thus obtain a high-quality initial solution. Second, we propose an approach based on a two-level configuration checking strategy and a tabu strategy to reduce the cycling problem. Third, we introduce a perturbation strategy and a vertex weighting strategy to help the algorithm be able to jump out of the local optimum effectively. Fourth, we combine the scoring functions *Cscore* and *Mscore* with the aforementioned strategies to propose effective methods for selecting vertices. These methods assist the algorithm in selecting vertices that are suitable for addition to or removal from the current candidate solution. Finally, we verify the performance advantages of the local search algorithm by comparing it with existing optimal heuristic algorithms on two sets of instances. The experimental results show that the algorithm exhibits better performance on two sets of classical instances.

## 1. Introduction

The minimum connected dominating set (MCDS) problem is a variant of the dominating set (DS) problem and is an NP-hard problem [1] that has many applications in various fields, such as ad hoc sensor networks [2], wireless sensor and actor networks [3], smartCloud vehicles [4], interconnection networks [5], and aviation networks [6]. In wireless sensor networks, finding the MCDS can be utilized to construct efficient coverage network structures where selected nodes can monitor the entire network area and maintain connectivity with each other. Thus, significant energy and other resource savings can be achieved by activating or maintaining the working state of only these critical nodes. MCDS can also serve as a virtual backbone network [7] for wireless sensor networks to establish reliable multi-hop routing paths. By selecting nodes in the connected dominating set as routers, the routing protocol design can be simplified, ensuring that any node in the network can transmit information to other nodes via at least one path. Additionally, constructing the MCDS helps simplify network management tasks such as centralized command dissemination, network configuration updates, or fault detection. Since the nodes in the dominating set can represent the entire network, interacting indirectly with all network nodes can be achieved by communicating with the nodes within the dominating set. Therefore, the MCDS plays a crucial role in practical applications, serving as an effective means to address many challenges in wireless sensor networks by optimizing resource utilization, enhancing network efficiency, and ensuring long-term operational capability.

Currently, numerous scholars have extensively researched the MCDS problem and proposed various algorithms to address it. These algorithms fall primarily into two categories: approximation algorithms and heuristic algorithms. Approximation algorithms compromise between computational complexity and exact solution attainment. While exact optimal solutions for many NP-hard problems are challenging to find within polynomial time, approximation algorithms can yield sub-optimal solutions that are close to optimal in a reasonable time frame. Heuristic algorithms, on the other hand, excel at finding satisfactory solutions quickly, making them especially suitable for tackling large and intricate problems. For NP-hard problems or other complex optimization challenges, the careful design of heuristic functions guides the search process, aiding in escaping local optimums and achieving convergence to high-quality solutions more rapidly. Consequently, heuristic algorithms are capable of generating near-optimal solutions within polynomial time. For approximate algorithms, Ruan et al. [8] proposed a new greedy approximation algorithm for solving the MCDS problem. The performance ratio is ln(Δ) + 2, where Δ represents the maximum degree of the input graph. Unlike all previously known one-step greedy algorithms with similar performance ratios, the greedy potential function of this algorithm is not supermodular. This implies that the performance analysis method of this new algorithm differs from previous methods based on supermodular potential functions, making it innovative. Sayaka et al. [9] presented a self-stabilizing algorithm applicable to wireless ad hoc networks and sensor networks aimed at finding MCDS while ensuring safe convergence properties. This algorithm is applied in network environments modeled by unit disk graphs to address the problem of constructing a virtual backbone network due to the absence of fixed infrastructure and centralized management. Yang et al. [10] proposed a greedy approximation algorithm for the MWCDS with labeling (Min-CDSL) problem. Min-CDSL introduces an additional constraint on top of the classical Min-CDS problem, requiring a reduction in the number of distinct labels in the dominating set while considering its size. This problem holds significant application value in wireless sensor networks and other communication networks, especially in constructing virtual backbone networks.

For heuristic algorithms, Jovanovic et al. [11] proposed a greedy starting point-based ACO algorithm and improved its global optimization capability in solving MCDSP through pheromone correction strategies and dynamic initialization strategies. Li et al. [12] introduced a greedy adaptive search algorithm to solve the MCDSP while employing tabu strategies to reduce cycling phenomena in local search. Wang et al. [13] proposed a variable-depth neighborhood search algorithm that effectively solves the MCDS problem by combining efficient neighborhood structures, tabu search, incremental evaluation methods, and search pruning strategies, demonstrating good solution quality and computational efficiency. Li et al. [14] proposed a greedy algorithm to solve the MCDS problem, employing a grid-checking strategy in the local search phase to help reduce the cycling problem while using a vertex adaptation mechanism to help the algorithm escape the local optimum. However, there are relatively few heuristic algorithms designed to tackle the MCDS problem. Therefore, we introduce a local search algorithm to address this problem.

Firstly, we propose three simplification rules to reduce the original graph, which effectively decrease the search space for our algorithm. Subsequently, we adopt a greedy strategy to construct an initial solution.

Furthermore, we propose a method based on a two-level configuration checking strategy and a tabu strategy to avoid cycling problems. Additionally, a perturbation strategy and vertex weighting strategy are introduced to help the algorithm effectively escape the local optimum when encountered.

Finally, we integrate the two-level configuration checking strategies, tabu strategy and perturbation strategy, as well as two scoring functions, *Cscore* and *Mscore*, to propose two effective vertex selection rules and a novel algorithm. Experimental results demonstrate that, compared to renowned algorithms such as the ant colony optimization algorithm (ACO) [11], the ant colony optimization algorithm with pheromone correction strategy (ACO + PCS) [11], the greedy randomized adaptive search procedure (GRASP) [12], the variable-depth neighborhood search algorithm (VDNS) [13], and the multi-start local search algorithm (MSLS) [14], the local search algorithm we proposed achieves superior results.

The remainder of this paper is structured as follows: Section 2 introduces relevant concepts; Section 3 provides a detailed description of the framework of the proposed algorithm and the specifics of the related strategies; Section 4 presents a comparative analysis of the experimental results of the algorithm proposed in this paper against other algorithms on two sets of instances; Section 5 offers a discussion and analysis of the experimental part; and finally, Section 6 concludes the paper.

## 2. Preliminaries

Give an undirected connected graph *G*(*V*, *E*), where *V* = {*v*_1_, *v*_2_, …, *v_n_*} denotes the vertex set, and *E* = {*e*_1_, *e*_2_, …, *e_m_*} denotes the edge set. *N*(*u*) represents the opened neighbor set of vertex *u*, denoted by *N*(*u*) = {*v* ∈ *V*|{*v*, *u*} ∈ *E*}. *deg*(*u*) = |*N*(*u*)| denotes the degree of vertex *u*. *N*[*u*] represents the closed neighbor set of vertex *u*, denoted as *N*[*u*] = *N*(*u*) ∪ {*u*}. Similarly, for a vertex subset *S*, *N*(*S*) $=\underset{v\in S}{⋃}N(v)\backslash S$, *N*[*S*] = *N*(*S*) ∪ *S*. The shortest distance between two vertices *v* and *u* in the graph is denoted as *dist*(*v*, *u*). The *i*-*dist* neighbor set of vertex *v* denotes *N_i_*(*u*) = {*v*|1 ≤ *dist*(*v*, *u*) ≤ *i*}, and *N_i_*[*u*] = *N_i_*(*u*) ∪ {*u*}. The *age*(*v*) value of vertex *v* is equal to the current iteration number when the state of vertex *v* changes (from dominated to undominated or from undominated to dominated). If vertex *v* is in the candidate solution, we say that vertex *v* dominates its neighbor set and itself. Here are some important definitions.

**Definition 1.**  *(dominating set, DS). In an undirected graph G(V, E), a dominating set is a subset S of V such that each vertex in V is either in S or adjacent to at least one vertex in S [15].*

**Definition 2.**  *(connected dominating set, CDS). Given an undirected connected graph G(V, E), a subset D is a connected dominating set if every pair of vertices in D is connected by a path where all vertices on the path are also in D [6].*

We employed two scoring functions, namely *Dscore* and *Mscore* [16], to guide the local search process. For a vertex *v*, *Dscore*(*v*) represents the number of vertices that change their status from non-dominated to dominated or vice versa among the neighbors of vertex *v* when vertex *v* is either added to or removed from the current candidate solution, as shown by Equation (1).

(1) $$Dscore(v)=\left\{\begin{array}{c} |N[D\backslash \{v\}]|-|N[D]|,\;\mathrm{if}\;v\in D\\ |N[D⋃\{v\}]\;\backslash \;N[D]|,\;\mathrm{if}\;v\notin D \end{array}\right.$$

where *N*[*D*] comprises the vertices within the current candidate solution *D* and their neighboring vertices; thus, *N*[*D*] can also be understood as the set of vertices dominated by the current candidate solution *D*. Similarly, *N*[*D*⋃{*v*}] is the set of vertices dominated by the current candidate solution *D* after adding vertex *v*. |*N*[*D*\{*v*}]| denotes the number of vertices dominated by the set *D*\{v}. According to Equation (1), *Dscore*(*v*) is non-positive when vertex *v* is added to the candidate solution, and *Dscore*(*v*) is non-negative when vertex *v* is removed from the candidate solution.

For vertex *v*, *Mscore*(*v*) signifies the number of neighboring vertices of *v* that are present in the candidate solution, as expressed by Equation (2).

(2) Mscore(v)= |N(v)∩D|

According to Formula (2), a vertex with a high *Mscore* means that it is surrounded by many neighbors that have been selected for the dominating set, and removing such a vertex may be more likely to cause the dominating set to lose connectivity or reduce coverage.

## 3. PCC^2^LS for the Minimum Connected Dominating Set Problem

Efficient heuristics play a pivotal role in guiding the search process. In Section 3.1, a deep dive into the construction of the initial solution is presented, offering detailed insights into the method employed. Following the construction of the initial solution, Section 3.2 elucidates the local search process aimed at further improving this initial solution. This section comprehensively explains the strategies used during this optimization phase, providing a thorough understanding of how the algorithm iteratively improves and enhances the candidate solutions.

### 3.1. Initial Solution Construction Procedure

The quality of the initial solution directly impacts the improvement results of the local search algorithm. To obtain a higher-quality initial solution, we propose an initialization construction procedure named InitConstructCDS, which is based on three simplification rules. In this process, a greedy strategy employing vertex-based *Dscore* values is utilized to construct the initial solution.

#### 3.1.1. Simplification Rules

In this subsection, we employ two operations—fixing and deletion—to simplify the original graph, thereby reducing the overall search space of the algorithm and obtaining a high-quality initial solution. The fixing and deletion operations we conduct ensure that certain vertices are guaranteed to be included in the optimal solution while also ensuring that certain other vertices will certainly not be part of the optimal solution. Next, I will introduce the three simplification rules in detail.

**Degree-1 Simplification Rule 1:** If there exists a vertical vertex *v* in graph *G*, i.e., *d*(*v*) = 1 with *N*(*v*) = {*u*}, then vertex *u* is fixed in the solution, and vertex *v* is subsequently deleted to simplify the original graph.

**Degree-2 Simplification Rule 2:** If graph *G* includes vertices *v*_1_, *v*_2_, and *u*, such that *N*(*v*_1_) = {*v*_2_, *u*} and *N*(*v*_2_) = {*v*_1_, *u*}, then vertex *u* is fixed in the current solution, and vertices *v*_1_ and *v*_2_ are subsequently deleted to simplify graph *G*.

**Degree-2 Simplification Rule 3:** If graph *G* contains vertices *v*_1_, *v*_2_, and *u*, where *N*(*v*_1_) = {*v*_2_, *u*} and *v*_2_ ∈ *N*(*u*), then the original graph *G* is simplified by deleting vertex *v*_1_.

**Figure 1:**
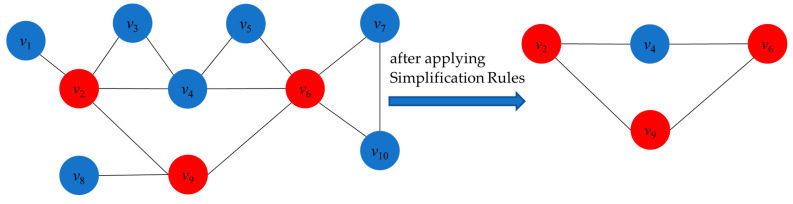
An example of applying simplification rules.

To better understand these three simplification rules, we use Figure 1 as an aid. Our goal is to find a connected dominating set of small size, so we want to add as few vertices as possible to the candidate solutions. Observe the following: *v*_1_ and *v*_8_ have degree 1 (*d*(*v*_1_) = *d*(*v*_8_) = 1), and their neighbors are *N*(*v*_1_) = {*v*_2_} and *N*(*v*_8_) = {*v*_9_}, respectively. If *v*_1_ and *v*_8_ are added to the solution, *v*_2_ and *v*_9_ must also be added to the solution in order to ensure the connectivity of the solution. On the other hand, directly adding *v*_2_ and *v*_9_ to the solution can equally dominate *v*_1_ and *v*_8_. Based on this observation, we propose **Degree-1 Simplification Rule 1**: fixing *v*_2_ and *v*_9_ in the solution and deleting *v*_1_ and *v*_8_ from the original graph. Furthermore, we consider that *v*_7_ and *v*_10_ have degree 2 (*d*(*v*_7_) = *d*(*v*_10_) = 2) and their neighbors *N*(*v*_7_) = {*v*_6_, *v*_10_} and *N*(*v*_10_) = {*v*_6_, *v*_7_}. Whether we choose to add *v*_7_ or *v*_10_ to the solution, *v*_6_ must be added to the solution at the same time in order to ensure the connectivity of the solution, again because *v*_6_ can dominate *v*_7_ and *v*_10_. Therefore, we propose **Degree-2 Simplification Rule 2**: fix *v*_6_ in the solution and remove *v*_7_ and *v*_10_ from the original graph. Finally, we observe that the degrees of *v*_3_ and *v*_5_ are 2 (*d*(*v*_3_) = *d*(*v*_5_) = 2), and their neighbors are *N*(*v*_3_) = {*v*_2_, *v*_4_} and *N*(*v*_5_) = {*v*_4_, *v*_6_}, where *v*_2_ is a neighbor of *v*_4_ and *v*_4_ is a neighbor of *v*_6_. When adding *v*_3_ and *v*_5_ to the solution, their neighboring vertices must be considered in order to ensure the connectivity of the solution. Whether we choose to add *v*_2_ or *v*_4_ and *v*_4_ or *v*_6_, we are able to dominate *v*_3_ and *v*_5_. Therefore, we propose **Degree-2 Simplification Rule 3**: Remove *v*_3_ and *v*_5_ from the original graph, as they can be effectively dominated by their neighboring vertices. By applying these rules, we obtain the simplified graph on the right-hand side. Next, we will look for a smaller connected dominating set in this simplified graph.

#### 3.1.2. InitConstructCDS Procedure

In this process, there are three main components: initialization, simplification, and construction. During the initialization phase, in line 1 of Algorithm 1, the *Dscore*(*v*) value of each vertex *v* ∈*V* is initialized to its degree plus one.

In the simplification phase, we apply three simplification rules to reduce the search space for the algorithm. Specifically, we follow a vertex sequence to assess whether a vertex should be fixed in the solution or removed from the graph. Vertices intended to be fixed in the solution are added to set *S*_1_, whereas those to be removed are appended to set *S*_2_. Thereafter, vertices from *S*_1_ are integrated into the candidate solution *D*, while those from *S*_2_ are incorporated into the set *REset*. At line 6, vertices from the *REset* are eliminated from the set *V*.

In the construction phase, we adopt a greedy strategy based on the *Dscore* values to generate an initial solution. More specifically, if the current candidate solution *D* is an empty set, at line 8, to break the randomness, we select an initial vertex with the biggest *Dscore* value from the *V* set. Subsequently, at line 9, the chosen vertex *v* is added to the set *D*. If the *D* is not empty, the algorithm iteratively selects vertices with the biggest *Dscore* values from the set *N*(*D*) and adds them to the *D* until a feasible solution is formed (lines 10–12). Finally, at line 13, once *D* forms a feasible solution, Algorithm 1 ends and returns the initial solution *D*.
**Algorithm 1**: InitConstructCDS( )**Input**: an undirected, connected graph *G*(*V*, *E*) **Output**: an initial connected dominating set *D* 1. Initialize *Dscore*(v) ← |*N*[*v*]| for all *v* in *V*; 2. *D* ← *REset*
←∅; 3. **while**
*v* ∈ *V*\(*D* ⋃ *REset*) in chronological order satisfying any simplification rules **then**
4.  obtain a fixed vertex set *S*_1_ and a deleted vertex set *S*_2_ based on simplification rule; 5.  *REset*
←
*REset*
⋃ *S*_2_ and *D* ←
*S*_1_; 6. *V*
←
*V*\*REset*; 7. **if** *D* = ∅ **then**
8.  *v* ← select one vertex with the biggest *Dscore* value from set *V*; 9.  *D* ← *D*∪{*v*}; 10. **while** *D* is not a *CDS* **then** 11.  *v* ← select one vertex with the biggest *Dscore* value from *N* 12.  *D* ← *D*∪{*v*}; 13. **return** *D*;

### 3.2. The Local Search Procedure in PCC^2^LS

This initial solution construction procedure may not yield a solution of very high quality; therefore, during the local search phase, we continuously optimize the initial solution by flipping vertices between inside and outside the solution through several heuristic strategies. To this end, we introduce the tabu strategy and two-level configuration checking strategy to assist the algorithm in mitigating the cycling problem. Secondly, we propose a perturbation strategy and a vertex weighting strategy to help the algorithm escape the local optimum. Lastly, when the current solution is disconnected, we propose a *CE* set to store the connected elements of the current solution, from which the algorithm can choose vertices to transform the current solution into a feasible one. Below, we will delve into the details of these associated heuristic strategies.

#### 3.2.1. Methods to Mitigate the Cycling Problem

Heuristic algorithms frequently revisit the most recently visited vertices during the solution process, leading to a waste of computational resources and affecting the performance of the algorithm. A complete solution to this problem has yet to be established. To effectively mitigate the cycling problem, we propose a hybrid approach combining a two-level configuration checking strategy with a tabu strategy to enhance algorithmic performance. The tabu strategy prevents vertices recently added to the solution from being immediately removed, while the two-level configuration checking strategy aids in selecting vertices to be added to the solution. Further elaboration on these two methods will be provided subsequently.

##### Tabu Strategy

The tabu strategy, originally conceived by Glover [17], is extensively applied in a variety of heuristic algorithms to tackle other combinatorial optimization problems, such as the maximum *s*-plex problem [18] and the minimum weight vertex cover problem [19].

The core mechanism of the tabu strategy consists of two key components: the tabu list and the tabu tenure. The tabu list is used to keep track of the vertices that have recently been added to the candidate solution, while the tabu tenure determines how long (or how many iterations) these vertices are prohibited from being removed from the solution again.

In the initial testing phase, we set the tabu tenure to 1. Then, in each round of iterations, vertices newly added to the candidate solution are immediately added to the tabu list, and these vertices are not allowed to be removed from the solution set in the next iteration. The purpose of this is to force the algorithm to explore vertices that have not been recently considered, thus altering the search trajectory and avoiding getting stuck in a cycle of visiting the same vertices over and over again.

By introducing the tabu strategy, the algorithm is able to traverse the solution space more efficiently, reducing ineffective repetitive computations and increasing the likelihood of finding a globally or near-globally optimal solution. Experimental results show that the tabu strategy can significantly improve the performance of the algorithm, effectively alleviate the cycle problem, and promote the convergence of the algorithm to higher-quality solutions.

##### Two-Level Configuration Checking Strategy

Configuration checking (CC) is a crucial concept where the configuration of a vertex holds significant physical meaning, representing the current state of relationships between a specific vertex and its neighboring vertices within a graph. In addressing the minimum vertex cover problem, Cai et al. [20] introduced the configuration checking strategy to circumvent local search algorithms becoming trapped in cycles characterized by repetitive visits to the same candidate vertices.

This strategy involves considering the configuration of a vertex when adding it to the candidate solution. Specifically, if the configuration of a vertex *v* remains unchanged since its last removal from the candidate solution, it is precluded from being re-added to the current candidate solution. This approach offers a conceptual framework that is both straightforward to implement and adaptable to various configuration-checking strategies, which are tailored to the specifics of the problem and algorithm at hand.

In our algorithm, we employ a two-level configuration checking strategy (CC^2^). Unlike the original configuration-checking strategy, CC^2^ considers the configurations of a vertex’s two-level neighbors. This entails updating the configurations of vertices *u* ∈ *N*_2_(*v*) whenever a vertex *v* is added to or removed from the candidate solution.

Similar to the original configuration checking strategy, a vertex is prevented from joining the candidate solution if its configuration remains unchanged since its last removal from the solution. In our implementation, we utilize the *ConfChange* value to represent the configuration status of each vertex. When *ConfChange*[*v*] = 1, it signifies that the configuration of vertex *v* has been altered, allowing the vertex to be added to the current candidate solution. Conversely, when *ConfChange*[*v*] = 0, it indicates that the configuration of vertex *v* has remained unchanged since its last removal from the candidate solution, thereby disallowing its addition to the current candidate solution.

**CC^2^_RULE 1**: At the beginning of the algorithm, *ConfChange*[*v*] is set to 1 for each vertex *v* ∈ V;

**CC^2^_RULE 2**: When vertex *v* is added to the candidate solution, *ConfChange*[*u*] is set to 1 for vertex *u* ∈ *N*_2_(*v*).

**CC^2^_RULE 3**: When vertex *v* is removed, *ConfChange*[*v*] is set to 0. For vertex *u* ∈ *N*_2_(*v*), *ConfChange*[*u*] is set to 1.

#### 3.2.2. Perturbation Strategy

After implementing the aforementioned strategy, we typically obtain some high-quality candidate solutions. However, these solutions may still be hindered by local optimization problems. To mitigate this limitation and broaden the algorithm‘s search space, we introduce a straightforward perturbation strategy aimed at identifying vertices of higher quality. This perturbation technique involves flipping certain vertices.

The perturbation strength serves as a crucial parameter in this strategy, significantly impacting its effectiveness. If the perturbation strength is too substantial, it may prompt the algorithm to restart, whereas too small a perturbation strength may fail to facilitate escape from the local optimum. To accommodate varying instance sizes, we set the number of perturbed vertices to 0.05 × |*V*|. Numerous experiments have demonstrated that this perturbation strength strikes a balance, neither excessively drastic nor overly feeble.

Thus, when the current solution becomes trapped in a local optimum, we greedily flip 0.05 × |*V*| vertices to facilitate escape. By perturbing a certain proportion of vertices in this manner, the algorithm can reassess and optimize the solution’s structure across a broader spectrum, thereby increasing the likelihood of escaping the local optimum. This strategy enhances the algorithm’s global search capability, ensuring the generation of high-quality approximate solutions even for NP-hard problems.

#### 3.2.3. Vertex Weighting Strategy

During the local search phase, optimizing the current solution involves continually flipping vertices both within and outside the solution. Hence, selecting which vertices to flip becomes paramount. We have observed that relying solely on the *Dscore* value for vertex selection easily traps the algorithm in local optima. To address this, we propose a vertex weighting strategy wherein the weight value assumes a pivotal role in guiding vertex selection. If a vertex remains undominated for an extended period, its weight value increases, influencing the overall vertex score and thereby prompting its flipping. Remarkably, the strategy boasts low complexity and straightforward implementation. Each vertex’s weight value, denoted as *Wscore*, undergoes updates based on predefined rules.

**Weight_Rule1**: In the initialization phase of the algorithm, the *Wscore* value for each vertex is initialized to 1;

**Weight_Rule 2**: In the local search phase, at the end of each iteration of the loop, the *Wscore* value for vertices that are not dominated is incremented by 1.

Based on the vertex weighting mechanism and the *Dscore* value, we introduce a novel scoring function termed *Cscore*, which amalgamates the individual *Dscore* value per vertex with the *Wscore* value. While the *Dscore* value facilitates dominance over more vertices, the *Wscore* value enables the algorithm to escape locally optimal solutions and explore alternative vertices. The formula for computing the *Cscore* value is presented in Equation (3).

(3) $$\mathrm{Cscore}(v)=\left\{\begin{array}{c} \sum_{u\in M1}Wscore(u)+Dscore(v),if\;v\notin D\\ -\sum_{u\in M2}Wscore(u)+Dscore(v),if\;v\in D \end{array}\right.$$

where *M*1 represents the subset of vertices within *N*[*v*] that are undominated, while *M*2 signifies the subset of vertices in *N*[*v*] that are solely dominated by vertex *v*. The vertex’s *Cscore* value incorporates both its *Dscore* and *Wscore* values.

#### 3.2.4. Connected Elements of Candidate Solutions

During the local search process, certain vertices are initially removed to disrupt the structure of the current candidate solution, thus producing an alternative candidate solution. These removed vertices result in the current candidate solution becoming disconnected, forming multiple connected components. Consequently, during the local search, efforts are made to identify vertices that can connect these disconnected components to each other.

To achieve this goal, we adopted the set *CE*. Set *CE* contains the connected elements of the current candidate solution. Assuming this candidate solution *S* = *S*_1_ ∪*S*_2_∪⋯∪*S_k_*, where *S*_1_, *S*_2_, ⋯, *S_k_*, are *k* connected components, *CE* = {*u*|Sum_A(*u*) = max{sum_A(*c*) = ∑*_j_*_= 1…*k*_A(*c*, *j*), *c* ∈ *V*\*S*}, *u* ∈ *V*\*S*, in which A(*c*, *j*) = 1 if *c* appears in *N*(*S_j_*), otherwise, A(*c*, *j*) = 0.

#### 3.2.5. Vertex Selection Method

During the local search phase, the algorithm continuously swaps vertices within the current solution with those outside it to refine the initial solution. Hence, selecting appropriate vertices for exchange becomes crucial. This ensures that the algorithm steers clear of the local optimum, thereby enhancing its overall performance. To achieve this, the algorithm integrates *Cscore*, *Mscore*, and all previously mentioned strategies to formulate the following rules for vertex selection:

**Add rule**: select the vertex *v* with the highest *Cscore* value and *ConfChange*[*v*] = 1. If multiple vertices share the highest *Cscore*, choose the one with the greatest age value.

**Remove rule**: set a fixed probability *fp*, generate a random probability *p*; if *p* < *fp*, choose a vertex with the largest *Cscore*; otherwise, choose a vertex with the smallest *Mscore*; if more than one satisfies the condition, choose a vertex with the largest *age*, but the chosen vertex is not in the tabu list.

#### 3.2.6. PCC^2^LS_Search Procedure

After constructing the initial solution using the *InitConstructCDS* procedure, the subsequent optimization in the local search procedure, PCC^2^LS_Search, aims to attain a smaller connected dominating set. To mitigate the cycling problem, we employ two two-level configuration checking strategies along with the tabu strategy. Additionally, it incorporates perturbation and vertex weighting strategies to guide the current candidate solution away from the local optimum. During vertex removal, solely selecting the vertex with the highest *Cscore* value exacerbates the cycling problem within the PCC^2^LS_Search process. Thus, the introduction of the *Mscore* function and noisy strategy [21] enhances vertex diversity, enabling the selection of vertices not previously considered in the candidate set. Moreover, a probability parameter, “*fp*”, decides whether to prioritize vertices with the largest *Cscore* or the minimum *Mscore* value. Finally, a candidate set, *CE*, is utilized to store the connected components of the current solution, ensuring that added vertices maintain solution feasibility. Based on these considerations, Algorithm 2 outlines the PCC^2^LS_Search procedure in detail.
**Algorithm 2**: PCC^2^_Local_Search( )**Input**: the maximum number of iterations *ITEM_NUM*; an undirected, connected graph *G*(*V*, *E*) **Output**: a local best connected dominating set *D’* **1.** D* ←*D* ←*InitConstructCDS*(G) and it ←1; **2.** Initialize ConfChange[*v*] ← 1 for each *v* in *V*; **3.** **While** it< ITEM_NUM **do** **4.**  **While** *D* is a *CDS* **do** **5.**    if |D|< |*D’*|**then** **6.**     D ← *D*; **7.**    v ← select one vertex from *D* according to **Remove_Rule**; **8.**   *D* ← D\{*v*}; **9.**   v ← select one vertex from *D* randomly; **10.**  *D* ← D\{*v*}; **11.**   for i=1 **to**
0.05 ∗|*V*| **12.**    v ← select one vertex from *D* according to **Remove_Rule**; **13.**    D ← D\{*v*}; **14.**  **While** *D* is not a *CDS* **do**
**15.**   update *CE*; **16.**    v ← select one vertex from *CE* according to **Add_Rule**; **17.**    D ← D∪{*v*}; **18.**    tabu_vertex ← *v*; **19.**  *it*++; **20.** **return** *D’*;

In the description of Algorithm 2, an initial solution is obtained in line 1 through the *InitConstructCDS*() function, and the number of iterative steps of the algorithm is initialized to 1. In line 2, the *ConfChange*(*v*) value of each vertex *v* ∈*V* is initialized to 1. In lines 5–6, when the current candidate solution *D* is a connected dominating set, *D* is compared with the local optimal solution *D’*. If *D* is better than *D’*, then *D’* is updated to *D*. Then, in lines 7–8, the algorithm systematically removes vertices from the *D* until it becomes infeasible. At this stage, the algorithm selects vertices from the candidate solutions according to the **Remove rule**. In lines 9–10, when *D* is not a connected dominating set, randomly remove a vertex from the candidate solution. In lines 12–13, in order to expand the search space of the algorithm, 0.05 × |*V*| vertices are removed from the candidate solutions according to the **Remove rule**. Next, in line 15, the set *CE* is updated. In lines 15–18, when *D* is not a connected dominating set, the algorithm adds some vertices to the candidate solution one by one until the candidate solution becomes a feasible connected dominating set. At this stage, the algorithm selects vertices from the set *CE* according to the **Add rule**. In line 18, add the vertices added to the candidate solution to the tabu list. Finally, in line 20, the locally best solution, *D’,* is returned.

## 4. Experimental Comparison

In this section, we conduct multiple experiments to assess the performance of our proposed algorithm, PCC^2^LS. The algorithm is evaluated across two sets of instances. Furthermore, we compare PCC^2^LS against six state-of-the-art algorithms: Greedy, ACO, ACO + PCS, GRASP, VDN, and MSLS. Subsequently, we provide descriptions of the two sets of instances and the four algorithms under comparison.

### 4.1. Instances Introduction

LPNMR’09 instances: The set of instances was tested at the 10th International Conference on Logic Programming and Nonmonotonic Reasoning. There are nine benchmark instances in this group, and the number of vertices in the instances ranges from 40 to 90, which is a relatively small size.

Common UDG instances: This set of benchmark instances was originally proposed by Jovanovic et al. [11] and is derived from ad hoc network clustering problems. There are 41 benchmark instances in this group, and the number of vertices in instances ranges from 80 to 400, which is a relatively large scale.

### 4.2. Comparison Algorithm

Greedy [5]: The algorithm constructs the global solution by selecting the locally optimal solution at each step, i.e., at each iteration step, the algorithm selects the vertices that are able to dominate more non-dominated vertices to be added to the dominating set and finally ensures that the generated dominating set remains connected.

ACO [11]: The design of the algorithm is based on a simple greedy algorithm proposed by Guha and Khuller [5]. The original single-step greedy algorithm tends to fall into local optimality because it is based on the vertex with the highest node degree as the starting point and constructs the solution by gradually adding the neighboring nodes that cover the largest number of undominated nodes.

ACO + PCS [11]: This algorithm is an improved ACO algorithm that guides the ant colony to search in a more probable direction by decreasing the pheromone concentration of those vertices that are not conducive to finding a better solution when the ACO algorithm encounters a stagnant situation. PCS dynamically updates the pheromone traces based on the nature of the current optimal solution, thus guiding the ant colony away from the local optimum and toward the global optimal solution.

GRASP [12]: The algorithm first constructs an initial candidate solution through the greedy principle and performs a localized search to continuously improve the quality of the solution based on it. Throughout the algorithmic framework, the search process is guided by a restricted candidate list (RCL) and a dynamically updated heuristic function, and duplicated decisions are circumvented with the help of a taboo table to ensure efficient search and good solution discovery.

VDN [13]: The algorithm is a variable-depth neighborhood search algorithm, which improves the search efficiency while limiting the search space through an efficient neighborhood structure and an incremental evaluation update technique.

MSLS [14]: The algorithm effectively improves the solving ability and convergence speed of the MCDS problem through well-designed heuristic strategies such as multiple restarts, vertex scoring mechanisms, configuration checking mechanisms, and vertex flipping mechanisms.

### 4.3. Experimental Environment and Parameters

The PCC^2^LS algorithm is coded in the C++ programming language and compiled with GNU g++ using the -O option. PCC^2^LS and MSLS run on an Intel Core i5-11400H processor clocked at 2.70 GHz. LPNMR’09 runs on a Genuine Intel Core 2 CPU 6600 at 2.40 GHz. ACO and ACO + PCS operate on a Genuine Intel Core 2 CPU E8500 running at 3.16 GHz. GRASP runs on an Intel Xeon CPU E7-4830 with a clock speed of 2.13 GHz. VNDS runs on an Intel i3 CPU clocked at 2.1 GHz. In order to obtain more high-quality candidate solutions for the proposed algorithm, we have conducted several experiments to finalize the parameters involved. The number of loop iterations is set to 50,000, the probability parameter *fp* is set to 0.5, and the number of perturbed vertices is set to 0.05 × |*V*|.

### 4.4. Experimental Results on LPNMR’09 Instances

Table 1 shows the experimental results of PCC^2^LS and other comparison algorithms on LPNMR’09 instances.

- The “problem” column shows the given number of vertices and edges in the input graph.
- The “LB” column shows the lower bound obtained by solving the Lagrangian dyadic problem using the subgradient method, which is used as a stopping criterion to verify that an optimal solution is found.
- The “LPNMR” shows the best results originally provided in the literature [11].
- The “average” row represents the average of the best values of the corresponding algorithm over all LPNMR’09 instances.
- The “Greedy” column shows the best solution obtained by the greedy algorithm.
- The “Avg” column represents the average solution obtained from 10 runs based on different randomly generated seeds.
- The “St.” column represents the standard deviation of the best solution obtained from 10 runs of the corresponding algorithm.
- The “Tim” column represents the average running time of 10 runs of the corresponding algorithm.

This way of presenting the results not only provides an evaluation of the performance of the algorithm on different instances but also analyzes the stability and efficiency of the algorithm by means of data such as mean, standard deviation, and running time.

Based on the results presented in Table 1, it is evident that our algorithm, PCC^2^LS, demonstrates superior performance compared to other algorithms, namely MSLS and GRASP, across both instances (70 × 250 and 90 × 600). Specifically, when considering the average (Avg) performance metric, PCC^2^LS outperforms the other algorithms consistently. Moreover, examining the standard deviation (St.), it is noteworthy that the PCC^2^LS algorithm consistently achieves a value of 0 on every instance. This implies that each run of the PCC^2^LS algorithm consistently yields the best solution, which is similar to the performance of the MSLS and GRASP algorithms. Additionally, in terms of runtime, the PCC^2^LS and MSLS algorithms generally exhibit faster execution compared to the other algorithms, indicating their efficiency in addressing this particular type of problem.

### 4.5. Experimental Results on Common UDG Instances

Table 2 presents the experimental outcomes of the PCC^2^LS algorithm alongside other comparative algorithms on common UDG instances. Each column retains the same definitions as in Table 1, where the “Min” column denotes the best solution achieved by each algorithm over 10 runs using distinct random seeds.

From Table 2, it can be observed that the greedy algorithm performs poorly on most of the problems compared to the other algorithms. The ACO and ACO + PCS algorithms obtain the best solution on only two instances (400_80_80) and (400_80_100). At the same time, the GRASP algorithm achieved the best solution in six instances. The VDNS algorithm found the same 18 best solutions within the 300 and 30 s time constraints, while the MSLS algorithm found the 28 best solutions. Although the PCC^2^LS algorithm failed to find the best solution in the (3000_400_210) instance, it achieved the best solution in all other instances. The PCC^2^LS algorithm significantly outperformed the other algorithms in terms of average solution. Therefore, in summary, the PCC^2^LS algorithm is more advantageous in terms of solution quality despite the longer solution time.

## 5. Discussion

### 5.1. Analysis of the Quality of the Solution

Based on the experimental results in Section 4.5, the PCC^2^LS algorithm consistently matches or outperforms compared algorithms on common UDG instances. For smaller graphs, these algorithms also yield competitive solutions, diminishing the algorithm’s advantage. However, on larger graphs, the algorithms demonstrate more pronounced benefits. To illustrate statistical findings effectively, we compare the best solutions obtained by six heuristic algorithms (Greedy, ACO, ACO + PCS, GRASP, VDN, and MSLS) with those from PCC^2^LS on identical examples.

**Figure 2:**
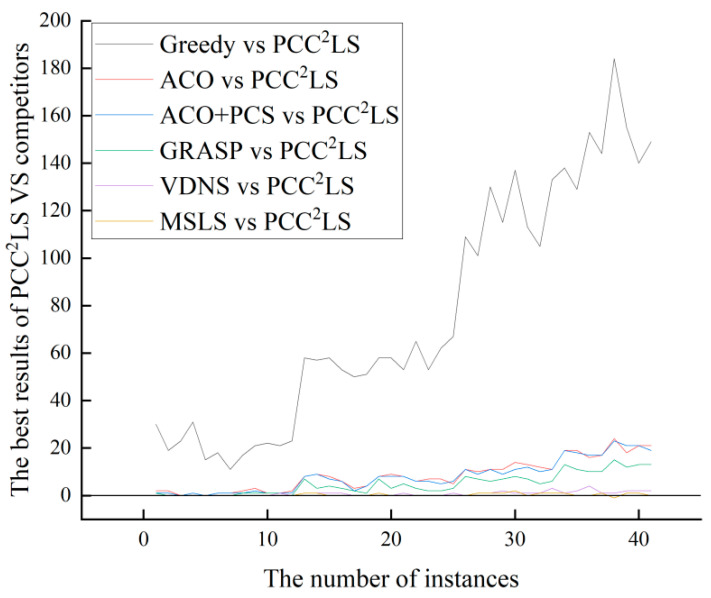
The comparison of ‘*BEST*’ values of greedy, ACO, ACO + PCS, GRASP, VDNS, MSLS, and PCC^2^LS on common UDG instances.

Figure 2 depicts the vertical axis as the difference between the best solution found by the comparison algorithm and that by the PCC^2^LS algorithm, labeled as *BEST* (comparison algorithm) – *BEST* (PCC^2^LS). The horizontal axis represents the instance number, where BEST (comparison algorithm) indicates the best value achieved by the comparison algorithm for each instance. A positive value on the vertical axis indicates that PCC^2^LS has found a superior solution. A value of 0 indicates that both algorithms found the same best solution. Conversely, a negative value indicates that the comparison algorithm performed better.

**Figure 3:**
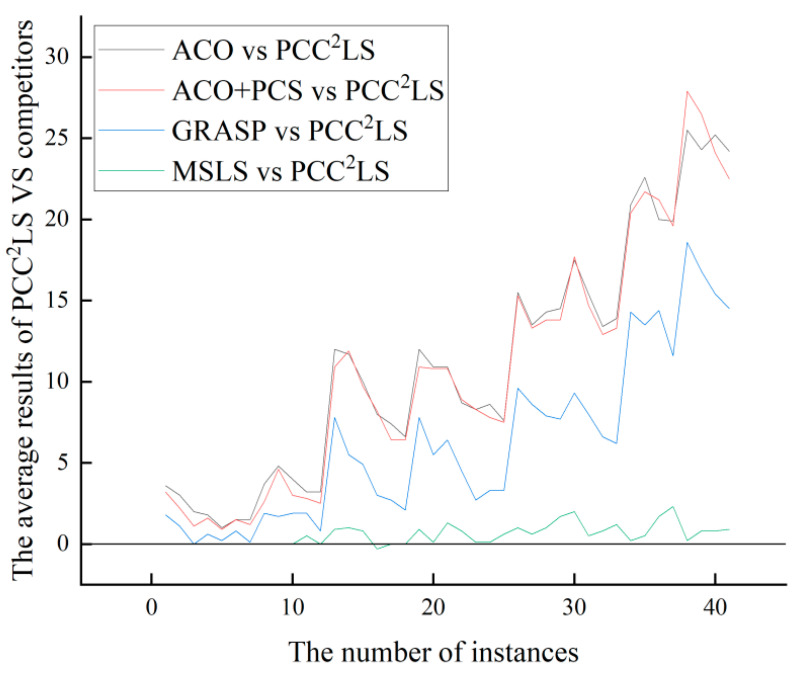
The comparison of ‘*Average*’ values of greedy, ACO, ACO + PCS, GRASP, VDNS, MSLS, and PCC^2^LS on common UDG instances.

From Figure 2 and Figure 3, it can be observed that the PCC^2^LS algorithm shows better performance in general, and the best and average solutions obtained by this algorithm outperform those obtained by other comparative algorithms. This is mainly due to the fact that three simplification rules are used to simplify the original graph in the initial solution construction stage, while the local optimum is handled in the local search stage by a perturbation strategy, a vertex penalization strategy, and a series of strategies to avoid the cyclic problem. Therefore, the PCC^2^LS algorithm has a significant advantage in minimizing the quality of the solution.

### 5.2. Analysis of Solution Times

**Figure 4:**
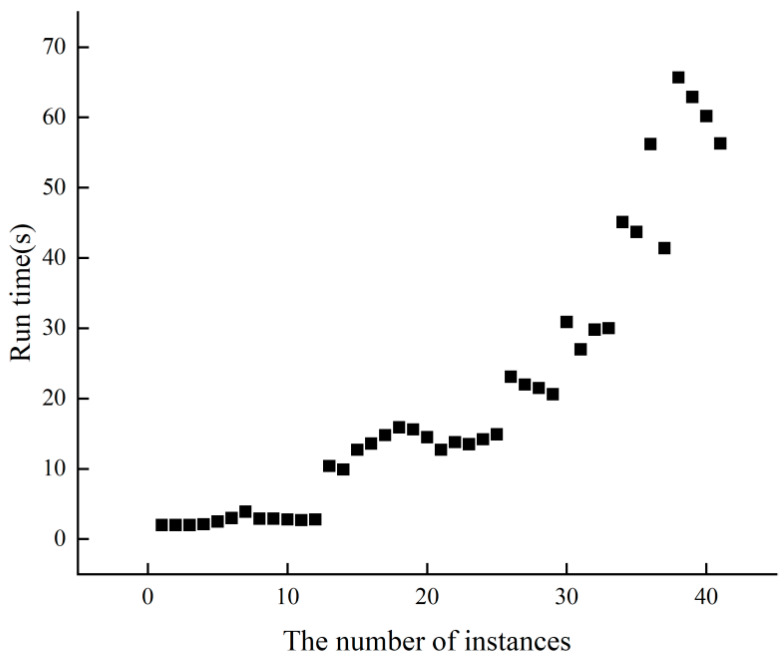
The run time of PCC^2^LS on common UDG benchmark instances.

Figure 4 displays the runtime performance of PCC^2^LS on classical common UDG benchmark instances. Each instance undergoes 10 runs, and the average time to achieve the best solution is calculated. It is evident that across 41 instances, the average time to reach the best solution does not exceed 70 s. Particularly for the initial 25 relatively smaller instances, PCC^2^LS efficiently identifies the best solution promptly.

### 5.3. Comparison of Computational Burden with Other Algorithms

**Figure 5:**
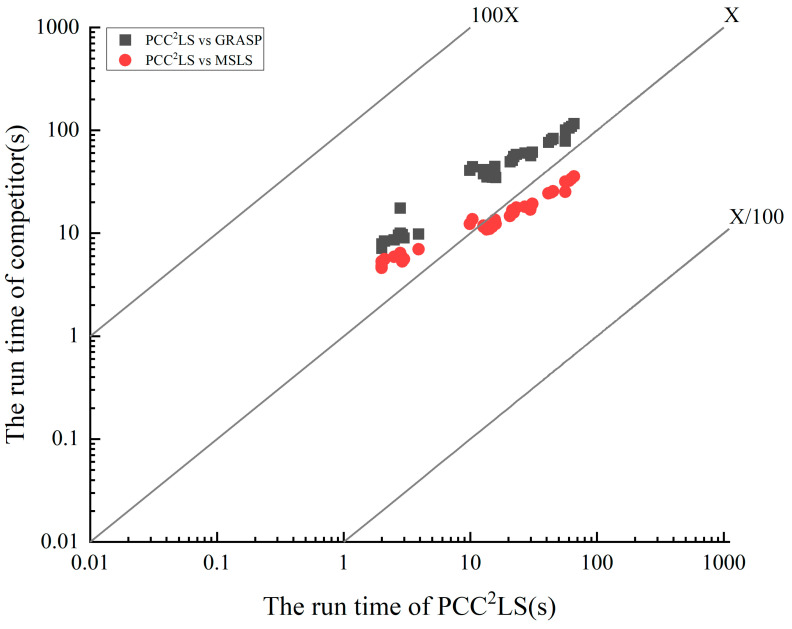
Comparing the time needed to find the best solution between PCC^2^LS and other algorithms on common UDG instances.

To visualize the difference in solution time of the PCC^2^LS algorithm compared to the comparison algorithms, Figure 5 vividly depicts the key findings from the data in Table 2. Given the uniqueness of the VDNS algorithm, which pursues the best solution within a predefined time bound, we focus on directly comparing the average solution time of the PCC^2^LS algorithm with that of the MSLS and GRASP algorithms, which are able to consistently find the best solution. In this graph, the horizontal axis represents the average time taken by the PCC^2^LS algorithm to reach the best solution for common UDG benchmark instances, while the vertical axis corresponds to the average time consumed by the MSLS and GRASP algorithms to achieve the same goal. Observing the illustrations, a notable feature is that all three algorithms are able to find the best solution for each instance in less than 100 s, demonstrating their superiority in terms of efficiency. Further, the relative position of the legend to the *X*-axis serves as an intuitive indicator for evaluating the performance of the algorithms: if the legend is above the *X*-axis, it implies that the PCC^2^LS algorithm occupies a significant advantage in terms of the solution speed; on the contrary, being below the *X*-axis indicates that the comparison algorithms are more efficient in this regard. Based on this analytical framework, Figure 5 clearly reveals that the average time to reach the best solution of the PCC^2^LS algorithm is generally shorter than that of the GRASP algorithm when dealing with common UDG instances. The comparison with the MSLS algorithm, on the other hand, reveals a more complex scenario–PCC^2^LS is sometimes faster and sometimes slightly less so, highlighting the relativity of algorithmic performance and the differences in advantages in specific scenarios.

Combining the discussions in Section 5.1, Section 5.2 and Section 5.3, when comparing these algorithms, the MSLS algorithm and the PCC^2^LS algorithm show better overall performance in solving the MCDS problem, especially in terms of the quality of the solution and computational efficiency. The PCC^2^LS efficiently improves the searching efficiency through the three simplification rules and the vertex weighting strategy, while the VDNS algorithm demonstrates efficiency and stability in most instances through the variable-depth neighborhood search and incremental evaluation methods. In contrast, although GRASP, ACO, ACO + PCS, and VDNS each have their own unique features, PCC^2^LS and MSLS perform better in terms of the quality of the computational burden and solution in most cases.

### 5.4. Sensitivity Analysis of Perturbation Strength

When studying the impact of perturbation strategies on algorithm performance, the sensitivity analysis of perturbation strength, as a core parameter, is crucial. The perturbation strength is directly related to the ability of the algorithm to jump out of the local optimal solution and the breadth of the global search, so the careful analysis of its impact on the quality of the solution and the convergence speed is a key step in optimizing the performance of the algorithm.

For concrete implementation, we first fixed the tabu tenure parameter to 1 and set a series of perturbation strength levels from 0.02 × |*V*| to 0.1 × |*V*| with a step size of 0.02 × |*V*|, which were gradually applied to our local search algorithm. For each selected value of perturbation strength, we run the algorithm 10 times at each intensity and record the best solution and average solution obtained in each run in Table 3.

By comparing the number of best solutions obtained and the average solution quality under different perturbation strengths, we can observe the specific impact of the perturbation strength on the performance of the algorithm. Ideally, a moderate perturbation strength can significantly improve the algorithm’s ability to jump out of the local optimal solution while avoiding the problem of degradation of solution quality or algorithmic efficiency caused by excessive perturbation. For example, when the perturbation strength is set to 0.05 × |*V*|, the algorithm shows better performance compared to other perturbation strengths and successfully finds smaller connected dominating sets.

### 5.5. Analysis of Performance Limitations and Worse Case

The PCC^2^LS algorithm demonstrates excellent performance in solving the MCDSP, particularly in terms of solution quality. Experimental verification shows that it consistently finds better solutions or achieves the best-known solutions compared to other state-of-the-art heuristic algorithms across a wide range of standard instances. However, despite PCC^2^LS’s efficiency and stability, it is essential to consider its performance limitations and potential worst-case scenarios.

Regarding processing time for large-scale graphs, PCC^2^LS typically finds the best solution within 30 s on most benchmark instances, performing exceptionally well in smaller-scale scenarios. Yet, as graph sizes increase further, although it can usually be completed within 70 s, it may approach or exceed acceptable waiting time thresholds for some real-world applications with stringent response-time requirements. This limits its applicability in real-time systems demanding extremely high response times.

In terms of parameter sensitivity, the algorithm’s performance hinges on various parameters such as iteration count, probability parameter (*fp*), and perturbation vertex ratio. Improper parameter settings can diminish algorithmic efficiency or degrade solution quality, necessitating meticulous parameter tuning tailored to specific problem instances, which can be time-consuming.

In worst-case scenarios, particularly with extremely dense graphs, finding a smaller set of connected dominants becomes exceptionally challenging. Each new vertex addition brings a significant number of neighboring vertices, potentially prolonging the algorithm’s exploration of the solution space or even preventing it from finding a satisfactory solution within the given time constraints.

However, for instances with unique structures (e.g., sparse graphs or those with specific fixed patterns), PCC^2^LS may not be optimized for specialized purposes, resulting in performance inferior to algorithms explicitly designed for these structures.

In summary, while PCC^2^LS generally performs well, it encounters performance limitations and challenges when faced with extreme conditions or specific problem types. Further research and optimization efforts are necessary to address these limitations effectively.

## 6. Conclusions

In this paper, a new local search algorithm named PCC^2^LS is proposed for solving the minimum connected dominating set problem. In the initial solution construction stage, three simplification rules are proposed to simplify the original graph. A combination of vertex weighting, perturbation, two-level configuration checking, and tabu strategies are used to avoid cyclic search and falling into local optimum. These methods ensure that the algorithm has good adaptability and stability in the face of different instance inputs and can find high-quality solutions even for NP-hard problems, which shows that the algorithm has good robustness. The design of the vertex weighting strategy makes the algorithm tend to select the vertices that have not changed their states for a long time, which increases the diversity and possibility of solution space exploration. Meanwhile, the perturbation strategy can effectively jump out of the local optimal solutions, which further promotes the generation of different solutions. Based on the above strategies and properties, a meta-heuristic local search algorithm PCC^2^LS is designed and compared with six state-of-the-art local search algorithms, namely, greedy, ACO, ACO + PCS, GRASP, VDNS, and MSLS, and the experimental results show that, on a number of benchmark instances, the proposed PCC^2^LS algorithm is able to find better solutions compared with the other compared algorithms or the known best solutions than the other comparative algorithms, showing higher solution quality. Specifically, the PCC^2^LS algorithm is able to find the known best solution on LPNMR’09 instances and obtain new upper bounds for 13 best solutions and 27 average solutions on common UDG instances. In terms of solution time, the PCC^2^LS algorithm is able to find the best solution in a relatively short time (less than 30 s on average) on most of the benchmark instances, especially for the smaller instances. Even for large-scale instances, the solution can be completed in a reasonable time (usually under 70 s) in most cases.

In the future, our proposed algorithmic framework can also be adapted to tackle other combinatorial optimization problems, including but not limited to the minimum vertex cover problem [22]**,** and the set-union knapsack problem [23,24]. Ongoing belief suggests that there is still room for improvement when addressing large-scale graph instances. For example, we can employ parallel local search algorithms based on CUDA (Compute Unified Device Architecture) [25] to solve some combinatorial optimization problems. We can utilize the CUDA framework to increase processing speed by distributing computationally intensive tasks to multiple cores of the GPU for parallel execution. As a result, we continually explore other strategies to further enhance the PCC^2^LS algorithm and effectively solve a greater number of large-scale graph instance problems. Meanwhile, we plan to extend the PCC^2^LS algorithm to more practical scenarios, such as social network analysis, gene regulatory networks in bioinformatics, and transportation network planning. By applying the algorithms in these specific domains, we aim to validate their generality and practicality. In addition, we will also customize the algorithm to improve it according to the needs of different applications in order to better solve practical problems.

## Figures and Tables

**Table 1:** Results of each algorithm on LPNMR’09 instances.

Problem	LB	LPNMR	Greedy	ACO			ACO + PCS			GRASP			VDNS		MSLS			PCC^2^LS		
				Avg	St.	Tim	Avg	St.	Tim	Avg	St.	Tim	Avg	Tim	Avg	St.	Tim	Avg	St.	Tim
40 × 200	5	5	10	5.8	0.6	3.2	5.3	0.45	4.1	***5***	0	7.1	***5***	<1	***5***	0	0.00	***5***	0	0.00
45 × 250	5	5	15	5.8	0.4	3.5	5.5	0.5	4.3	***5***	0	8.7	***5***	<1	***5***	0	0.00	***5***	0	0.00
50 × 250_1	6	8	15	8.1	0.54	4.8	8	0	6.1	***7***	0	9.8	***8***	<1	***7***	0	0.00	***7***	0	0.01
50 × 250_2	6	7	17	7.5	0.5	5	7.1	0.3	6.5	***7***	0	9.9	***7***	<1	***7***	0	0.01	***7***	0	0.00
55 × 250	6	8	20	8.8	0.98	5.6	8.3	0.45	7.3	***8***	0	9.8	***8***	<1	***8***	0	0.01	***8***	0	0.01
60 × 400	5	7	15	***7***	0	6.1	***7***	0	9.1	***7***	0	12.7	***7***	<1	***7***	0	0.01	***7***	0	0.00
70 × 250	11	13	32	14.2	0.74	11	13.9	1.04	13.5	***12***	0	11.5	13	<1	***12***	0	0.00	***12***	0	0.00
80 × 500	7	9	20	10	0.44	12	9.8	0.4	16.9	***9***	0	15.1	***9***	<1	***9***	0	0.00	***9***	0	0.01
90 × 600	7	10	19	10.9	0.83	14	10.6	1.01	17.3	***9***	0	17.2	10	<1	***9***	0	0.01	***9***	0	0.01
average	6.4	8.0	18.1	8.7			8.3			7.7			8.0		7.7			7.7		

**Table 2:** Results of each algorithm on common UDG instances.

Problem	LB	Greedy	ACO		ACO + PCS		GRASP			VDNS		MSLS			PCC^2^LS		
			Min	Avg	Min	Avg	Min	Avg	Tim	300 s	30 s	Min	Avg	Tim	Min	Avg	Tim
400_80_60	12	48	20	21.6	19	21.2	19	19.8	7.1	***18***	***18***	***18***	18	4.6	***18***	18	2
400_80_70	10	33	16	17	15	16.2	***14***	15.1	7.8	***14***	***14***	***14***	14	4.8	***14***	14	2
400_80_80	8	35	***12***	14	***12***	13.1	***12***	12	7.9	***12***	***12***	***12***	12	5.3	***12***	12	2
400_80_90	7	41	11	11.8	11	11.6	***10***	10.6	8.4	***10***	***10***	***10***	10	5.6	***10***	10	2.1
400_80_100	6	23	***8***	9	***8***	8.9	***8***	8.2	8.6	***8***	***8***	***8***	8	5.9	***8***	8	2.5
400_80_110	6	25	8	8.5	8	8.5	***7***	7.8	9	***7***	***7***	***7***	7	5.6	***7***	7	3
400_80_120	5	17	7	7.5	7	7.2	***6***	6.1	9.8	***6***	***6***	***6***	6	7	***6***	6	3.9
600_100_80	14	38	23	24.7	22	23.6	22	22.9	9	***21***	***21***	***21***	21	5.8	***21***	21	2.9
600_100_90	14	40	22	23.8	21	23.6	20	20.7	9.7	***19***	***19***	***19***	19	5.3	***19***	19	2.9
600_100_100	11	38	17	20	17	19	17	17.9	10	***16***	***16***	***16***	16	6.1	***16***	16	2.8
600_100_110	10	35	15	17.2	15	16.8	15	15.9	9.6	15	15	***14***	14.5	6.1	***14***	14	2.7
600_100_120	9	36	15	16.2	14	15.5	***13***	13.8	17.5	***13***	***13***	***13***	13	6.4	***13***	13	2.8
700_200_70	26	96	46	50.7	46	49.6	45	46.5	44.2	39	39	39	39.6	13.7	***38***	38.7	10.4
700_200_80	22	89	41	43.7	41	43.9	35	37.5	40.8	33	33	33	33	12.3	***32***	32	9.9
700_200_90	18	84	34	36	33	35.7	30	30.9	37.8	27	27	***26***	26.8	11.8	***26***	26	12.7
700_200_100	16	75	28	30.8	28	31	25	25.8	35.2	23	23	***22***	22.5	11.2	***22***	22.8	13.6
700_200_110	14	70	23	27.4	22	26.4	22	22.7	35.6	***20***	***20***	***20***	20	11.5	***20***	20	14.8
700_200_120	12	68	21	23.6	21	23.4	18	19.1	34.7	***17***	***17***	***17***	17	12.3	***17***	17	15.9
1000_200_100	26	96	46	50.7	46	49.6	45	46.5	44.5	39	39	39	39.6	13.5	***38***	38.7	15.6
1000_200_110	22	92	43	44.9	42	44.8	37	39.5	41.5	***34***	***34***	***34***	34.1	11.7	***34***	34	14.5
1000_200_120	20	82	37	39.9	37	39.8	34	35.4	41.5	30	30	***29***	30.3	11.5	***29***	29	12.7
1000_200_130	18	91	32	34.7	32	34.9	29	30.5	38.4	***26***	***26***	***26***	26.8	11.5	***26***	26	13.8
1000_200_140	16	76	30	31.3	29	31.3	25	25.7	35.3	***23***	***23***	***23***	23.1	10.9	***23***	23	13.5
1000_200_150	15	83	28	29.6	26	28.8	23	24.3	35.8	***21***	***21***	***21***	21.1	11	***21***	21	14.2
1000_200_160	14	86	24	26.6	25	26.5	22	22.3	35	20	20	***19***	19.6	11.5	***19***	19	14.9
1500_250_130	33	158	60	64.5	60	64.3	57	58.6	58.3	***49***	***49***	***49***	50	17.7	***49***	49	23.1
1500_250_140	30	144	53	57.2	52	57	50	52.3	55.6	44	44	44	44.3	15.9	***43***	43.7	22
1500_250_150	27	170	51	54.9	51	54.4	46	48.5	51	41	41	41	41.6	16.7	***40***	40.6	21.5
1500_250_160	25	151	47	50.5	45	49.8	43	43.7	49.4	38	38	37	37.7	14.7	***36***	36	20.6
2000_300_200	28	178	55	58.6	52	58.8	49	50.4	61.2	42	43	43	43.1	19.3	***41***	41.1	30.9
2000_300_210	26	151	51	53.5	50	52.8	45	46.1	60.1	39	39	***38***	38.6	18.1	***38***	38.1	27
2000_300_220	24	140	47	48.9	45	48.4	40	42.1	59	36	36	36	36.3	16.9	***35***	35.5	29.8
2000_300_230	23	166	44	47.5	44	46.9	39	39.8	56.4	36	36	34	34.8	18.1	***33***	33.6	30
2500_350_200	41	198	79	82	79	81.5	73	75.4	83.1	61	61	61	61.3	25.6	***60***	61.1	45.1
2500_350_210	39	185	75	79.1	74	78.2	67	70	80.8	58	58	***56***	57	24.8	***56***	56.5	43.7
2500_350_220	36	205	68	72.6	69	73.8	62	67	78.1	56	56	***52***	54.3	25.2	***52***	52.6	56.2
2500_350_230	34	193	66	69.2	66	68.9	59	60.9	76.1	50	50	50	51.6	24.4	***49***	49.3	41.4
3000_400_210	51	259	99	101.6	98	104	90	94.7	116.1	76	77	***74***	76.3	35.7	75	76.1	65.7
3000_400_220	48	225	88	95.4	91	97.6	82	87.9	108.9	72	73	71	71.9	33.8	***70***	71.1	62.9
3000_400_230	45	205	86	91.4	86	90.3	78	81.6	105.2	67	68	66	67	32.3	***65***	66.2	60.2
3000_400_240	42	210	82	85.8	80	84.1	74	76.1	100.4	63	65	***61***	62.5	31.7	***61***	61.6	56.3
Average	22	108.2	40.4	43.3	40	43	36.8	38.4		32.7	32.8	32.2	32.7		31.9	32.1	

**Table 3:** Sensitivity analysis of perturbation strength in common UDG instances.

Problem	0.02 × |*V*|		0.04 × |*V*|		0.05 × |*V*|		0.06 × |*V*|		0.08 × |*V*|		0.1 × |*V*|	
	Min	Avg	Min	Avg	Min	Avg	Min	Avg	Min	Avg	Min	Avg
400_80_60	***18***	18	***18***	18	***18***	18	***18***	18	***18***	18	***18***	18
400_80_70	***14***	14	***14***	14	***14***	14	***14***	14	***14***	14	***14***	14
400_80_80	***12***	12	***12***	12	***12***	12	***12***	12	***12***	12	***12***	12
400_80_90	***10***	10	***10***	10	***10***	10	***10***	10	***10***	10	***10***	10.8
400_80_100	***8***	8	***8***	8	***8***	8	***8***	8	***8***	8	9	9
400_80_110	***7***	7	***7***	7	***7***	7	***7***	7	***7***	7	9	9
400_80_120	***6***	6	***6***	6	***6***	6	***6***	6	***6***	6	7	7.9
600_100_80	***21***	21	***21***	21	***21***	21	***21***	21	***21***	21	***21***	21
600_100_90	***19***	19	***19***	19	***19***	19	***19***	19	***19***	19	***19***	19
600_100_100	***16***	16	***16***	16	***16***	16	***16***	16	***16***	16	***16***	16
600_100_110	***14***	14	***14***	14	***14***	14	***14***	14.5	***14***	14	***14***	14
600_100_120	***13***	13	***13***	13	***13***	13	***13***	13	***13***	13	***13***	13
700_200_70	39	39.9	***38***	38.8	***38***	38.7	***38***	38.1	***38***	38	39	39
700_200_80	32	32.9	***32***	32	***32***	32	***32***	32	***32***	32	***32***	32.9
700_200_90	***26***	26	***26***	26	***26***	26	***26***	26.5	27	27	27	27
700_200_100	***22***	22.6	***22***	22.2	***22***	22.8	***22***	22.7	23	23	23	23
700_200_110	***20***	20	***20***	20	***20***	20	***20***	20	***20***	20	21	21.9
700_200_120	***17***	17	***17***	17	***17***	17	18	18	18	18	20	20.8
1000_200_100	39	39.9	***38***	38.8	***38***	38.7	***38***	38.1	***38***	38	39	39
1000_200_110	***34***	34.1	***34***	34	***34***	34	***34***	34	***34***	34.4	***34***	34.1
1000_200_120	***29***	29.6	***29***	29	***29***	29	***29***	29	***29***	29.8	30	30.2
1000_200_130	***26***	26	***26***	26	***26***	26	***26***	26.5	***26***	26.6	***26***	26.1
1000_200_140	***23***	23	***23***	23	***23***	23	***23***	23	***23***	23	***23***	23.9
1000_200_150	***21***	21.1	***21***	21	***21***	21	***21***	21	***21***	21	22	22
1000_200_160	***19***	19	***19***	19	***19***	19	20	20	20	20	21	21
1500_250_130	50	51.3	***49***	49.5	***49***	49	***49***	49	***49***	49.1	50	50
1500_250_140	44	44.7	***43***	43.7	***43***	43.7	***43***	43.4	***43***	43.9	44	44.8
1500_250_150	41	41.1	***40***	40.7	***40***	40.6	***40***	40.4	***40***	40.9	42	42
1500_250_160	***36***	36.1	***36***	36	***36***	36	***36***	36	***36***	36	***36***	36.5
2000_300_200	42	42.4	***41***	41.4	***41***	41.1	***41***	41.5	43	43	43	43.9
2000_300_210	39	39	***38***	38.1	***38***	38.1	39	39	39	39.5	39	39.8
2000_300_220	***35***	35.7	***35***	35.1	***35***	35.5	***35***	35.4	36	36.1	36	36.5
2000_300_230	***33***	33.7	***33***	33.4	***33***	33.6	***33***	33.7	35	35	34	34.8
2500_350_200	62	62.3	***60***	61.2	***60***	61.1	***60***	60.4	***60***	60.8	62	62.6
2500_350_210	56	57.3	***55***	56.5	56	56.5	56	56	56	56.8	57	58.4
2500_350_220	52	53.7	52	52.9	52	52.6	***51***	52.4	53	53.9	55	55.7
2500_350_230	50	50.6	***49***	49.7	***49***	49.3	***49***	49.6	50	51	52	52.6
3000_400_210	78	78.7	***75***	76.4	***75***	76.1	***75***	75.6	***75***	75.4	76	77
3000_400_220	73	73.6	72	72	***70***	71.1	***70***	71.1	***70***	70.8	72	72.6
3000_400_230	67	67.6	66	66.4	***65***	66.2	**66**	66	66	66.6	68	68.1
3000_400_240	63	63.9	***61***	61.7	***61***	61.6	***61***	61.5	62	62.8	64	64.4
Number of best solutions	25		38		***39***		36		28		14	

## Data Availability

The original contributions presented in the study are included in the article, further inquiries can be directed to the corresponding authors.

## References

[1] Garey M., Johnson D.S. (1979). Computers and Intractability, a Guide to the Theory of NP—Completeness.

[2] Guo Y., Zhang Y., Mi Z., Yang Y., Obaidat M. (2019). Distributed task allocation algorithm based on connected dominating set for WSANs. Ad Hoc Netw..

[3] Thai M., Tiwari R., Du D.Z. (2008). On construction of virtual backbone in wireless ad hoc networks with unidirectional links. IEEE Trans. Mob. Comput..

[4] Chinnasamy A., Sivakumar B., Selvakumari P., Suresh A. (2019). Minimum connected dominating set based RSU allocation for smartCloud vehicles in VANET. Clust. Comput..

[5] Guha S., Khuller S. (1998). Approximation algorithms for connected dominating sets. Algorithmica.

[6] Li J., Wen X., Wu M., Liu F., Li S. (2019). Identification of key nodes and vital edges in aviation network based on minimum connected dominating set. Phys. A Stat. Mech. Its Appl..

[7] Wang Y., Wang W., Li X. (2005). Distributed low-cost backbone formation for wireless ad hoc networks. Proceedings of the 6th ACM International Symposium on Mobile Ad Hoc Networking and Computing (MobiHoc ‘05).

[8] Ruan L., Du H., Jia X., Wu W., Li Y., Ko K. (2004). A greedy approximation for minimum connected dominating sets. Theor. Comput. Sci..

[9] Sayaka K., Hirotsugu K. (2012). A self-stabilizing 6-approximation for the minimum connected dominating set with safe convergence in unit disk graphs. Theor. Comput. Sci..

[10] Yang Z., Shi M., Wang W. (2020). Greedy approximation for the minimum connected dominating set with labeling. Optim. Lett..

[11] Jovanovic R., Tuba M. (2013). Ant colony optimization algorithm with pheromone correction strategy for the minimum connected dominating set problem. Comput. Sci. Inf. Syst..

[12] Li R., Hu S., Gao J., Zhou Y., Wang Y., Yin M. (2017). GRASP for connected dominating set problems. Neural Comput. Appl..

[13] Wang L., Zhou T., Wu X. (2016). Variable-depth neighborhood search algorithm for the minimum-connected dominating-set problem. Sci. Sin. Inf..

[14] Li R., Hu S., Liu H., Li R., Ouyang D., Yin M. (2019). Multi-start local search algorithm for the minimum connected dominating set problems. Mathematics.

[15] Liedloff M. (2008). Finding a dominating set on bipartite graphs. Inf. Process. Lett..

[16] Niu D., Liu B., Yin M., Zhou Y. (2023). A new local search algorithm with greedy crossover restart for the dominating tree problem. Expert Syst. Appl..

[17] Glover F. (1989). Tabu Search—Part I. ORSA J. Comput..

[18] Zhou Y., Hao J. (2017). Frequency-driven tabu search for the maximum *s*-plex problem. Comput. Oper. Res..

[19] Cai S., Li Y., Hou W., Wang H. (2018). Towards faster local search for minimum weight vertex cover on massive graphs. Inf. Sci..

[20] Cai S., Su K., Sattar A. (2011). Local search with edge weighting and configuration checking heuristics for minimum vertex cover. Artif. Intell..

[21] Ma Z., Fan Y., Su K., Li C., Sattar A. Local search with noisy strategy for minimum vertex cover in massive graphs. Proceedings of the 14th Pacific Rim International Conference on Artificial Intelligence.

[22] Gu J., Guo P. (2021). PEAVC: An improved minimum vertex cover solver for massive sparse graphs. Eng. Appl. Artif. Intell..

[23] Wei Z., Hao J. (2021). Kernel based tabu search for the Set-union Knapsack Problem. Expert Syst. Appl..

[24] Wei Z., Hao J. (2021). Multistart solution-based tabu search for the Set-Union Knapsack Problem. Appl. Soft Comput..

[25] Sonuç E., Özcan E. (2024). CUDA-based parallel local search for the set-union knapsack problem. Knowl.-Based Syst..

